# On-chip wavefront shaping with dielectric metasurface

**DOI:** 10.1038/s41467-019-11578-y

**Published:** 2019-08-07

**Authors:** Zi Wang, Tiantian Li, Anishkumar Soman, Dun Mao, Thomas Kananen, Tingyi Gu

**Affiliations:** Department of Electrical and Computer Engineering, University of Delaware, Newark, DE 19711 USA

**Keywords:** Engineering, Sub-wavelength optics, Metamaterials

## Abstract

Metasurfaces can be programmed for a spatial transformation of the wavefront, thus allowing parallel optical signal processing on-chip within an ultracompact dimension. On-chip metasurfaces have been implemented with two-dimensional periodic structures, however, their inherent scattering loss limits their large-scale implementation. The scattering can be minimized in single layer high-contrast transmitarray (HCTA) metasurface. Here we demonstrate a one-dimensional HCTA based lens defined on a standard silicon-on-insulator substrate, with its high transmission (<1 dB loss) maintained over a 200 nm bandwidth. Three layers of the HCTAs are cascaded for demonstrating meta-system functionalities of Fourier transformation and differentiation. The meta-system design holds potential for realizing on-chip transformation optics, mathematical operations and spectrometers, with applications in areas of imaging, sensing and quantum information processing.

## Abstract (web summary)

Metasurfaces can be programmed for a spatial transformation of the wavefront, allowing on-chip optical signal processing. Here, the authors demonstrate a one-dimensional high-contrast transmitarray metasurface-based lens on SOI substrate and demonstrate functionalities of Fourier transformation and differentiation.

## Introduction

Metasurfaces are arrays of subwavelength structures capable of imposing a localized and spatially varying phase shift onto the transmitted or reflected electromagnetic (EM) wave^1–3^. Gradient variations of nanostructures in a subwavelength thin layer are capable of manipulating an out-of-plane EM wave in free space, leading to numerous applications from simple components of miniaturized flat lenses^4–8^ and holograms^9–11^, to more complicated systems of analog^12,13^ signal processing and spectrometers^14,15^. Metamaterials have also been used for manipulating in-plane waves^16–28^. Periodic structures, such as dielectric photonic crystals^29^ and metallic hyperbolic metasurfaces^30^, control the delay and momentum of in-plane light propagation. The inverse design method significantly reduces the footprint of functional simple components^19,20^. Optimization toward functions with multi-input/output for parallel signal processing can be of a significant computational cost, as the inverse design is numerically driven. Gradient varying on-chip metasurface based on plasmonics^23,24^ or dielectric metamaterials^16–18,25–28^ have been demonstrated for the on-chip lens. Compared with the two-dimensional metasurface-based image processors^12^, the on-chip meta-system can operate without an alignment step, but limited to process one-dimensional (1D) data represented through the wavefront in *x*–*y* plane^16,23,31,32^. The insertion loss of reported on-chip metasurfaces (ranges from a few dB to tens of dB)^16,23^ is not suitable to meet the requirements for standard passive optical components (<1 dB), and their small critical dimensions (<100 nm) makes them incompatible with foundry process and more vulnerable to fabrication related geometric distortions (Supplementary Table 1).

As an analogy of electronic circuits, current photonic microsystem’s complexity and capability^33–35^ will be eventually limited by the individual components’ size and energy consumption. In this work, we explore parallel signal processing through on-chip 1D high-contrast transmitarray (HCTA). By slightly adjusting the dimension of the void slots defined in the device layer, we can control the coherent inference of the parallelly transmitted wavefront with low loss. We design, fabricate and characterize the on-chip metasurface lens (metalens) made of HCTA. The 1D HCTAs maintain less than 1 dB insertion loss across the S, C, and L telecommunication bands (>200 nm bandwidth) while maintaining a good tolerance to fabrication variation. The HCTA exhibits low insertion loss, which is feasible for parallel and multi-stage on-chip signal processing. Based on the 1D HCTA design, we experimentally demonstrate ultra-short, low loss and broadband mode size convertors and metasystems performing Fourier transform and spatial differentiation. With minimal feature size of 140 nm, the 1D HCTA is compatible with current deep UV photolithography technique used in silicon photonics foundry, and thus feasible for large-scale silicon photonic computational chips operating at the speed of light.

## Results

### Low loss 1D on-chip HCTAs

**Fig. 1:**
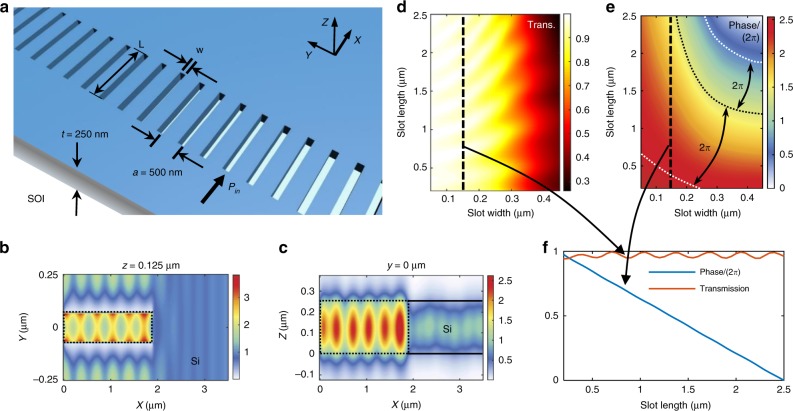
The design principle of low loss on-chip HCTAs. **a** A schematic view of 1D HCTA with a lattice constant *a* = 500 nm, defined on SOI substrate. It can impose localized phase shift on the electromagnetic wave traveling in plane. **b** Typical top (*x*–*y* plane) and **c** side (*x*–*z* plane) view of the intensity profile |*E*_*y*_|^2^ near the air slot. The dotted lines show the outline of the slot and the solid lines show the outline of silicon slab. **d** Simulated amplitude and **e** phase retardation of the transmission as a function of the slot width and length. The wavelength of the input light is 1550 nm. **f** The simulated amplitude and phase of the transmission versus slot length, fixing the slot width to be 140 μm

The designed HCTAs are 1D rectangular etched slot arrays defined in the silicon-on-insulator (SOI) substrate (Fig. 1a). Here we fix the lattice constant (*a*) of the HCTAs to be less than half of the wavelength (500 nm), and sweep the length (*L*) and width (*w*) to get the transmission and phase shift of the HCTA. The large refractive index contrast between silicon and silicon dioxide (>2) allows for a 2π phase shift to be achieved with high transmission^36^. The phase shift is introduced through the wave number differences in the slot and slab waveguides^37^ (Supplementary Figure 1). The optical mode profiles of the transmitted transverse electric (TE) wave tunneling through the slot are illustrated in Fig. 1b, c. The transmission and the phase shift can be controlled by varying the width and length of the slots (Fig. 1d, e). A phase shift from 0 to 2π combined with a transmission larger than 94% can be simultaneously achieved by fixing the slot width to be 0.14 µm while varying the slot length from 0.2 to 2.5 μm (Fig. 1f). It is noted that with a wider slot, a 2π phase shift can be achieved with a smaller slot length variation, but the transmission drops dramatically (Fig. 1d).

### Compact HCTA metalens

Here we use a gradient varying HCTA for on-chip wavefront control. The 1D HCTA along the *y* direction imposes a space-dependent phase shift on the impinging light (TE polarized) along the x direction. The phase shift of the transmitted wave is defined in the following equation for achieving the on-chip wave focusing:

1 $$\phi \left( y \right) = \frac{{2\pi }}{{\lambda _{\mathrm{d}}}}n_{{\mathrm{eff}}}\left( {f - \sqrt {f^2 + y^2} } \right)$$

where *λ*_d_ is the design wavelength in free space, *n*_eff_ is the effective refractive index of the guided light confined in the silicon slab, and *f* is the focal length. Here we fix the lattice constant (*a* = 500 nm) and slot width (*w* = 140 nm) for ensuring high transmission, and vary the slot length along the *y* direction for achieving the desired wavefront.

**Fig. 2:**
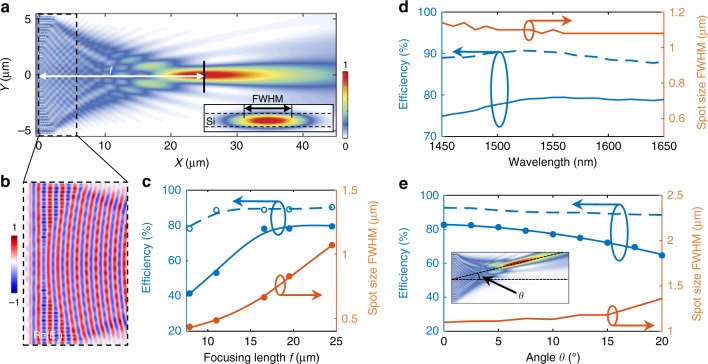
HCTA based on-chip metalens. **a** In-plane light distribution of |**E**_**y**_|^2^ in the middle of the silicon slab with incident light parallel to its optical axis. Inset: cross-sectional view of the light distribution on the focal plane, with the minimal spot size marked as FWHM. **b** Simulated electric field distribution, **E**_**y**_, in the region highlighted by a dashed box in **a**. **c** Simulated focusing efficiency (solid dots), transmission (empty circles) and FWHM versus focal length as marked in **a**. The curves are eye guides. The lens dimension is fixed at 10 µm with varying lens design. **d** Focusing efficiency, transmission and the FWHM of the metalens with a focal length of 25 μm. **e** Focusing efficiency, transmission and the FWHM of the metalens with a focal length of 25 μm at oblique incidence angle from 0^o^ to 20^o^. Inset: In-plane light distribution of |**E**_**y**_|^2^ at an oblique incident angle of 7.5°

The analytically designed metalens is then numerically evaluated (see method) (http://www.lumerical.com/tcad-products/fdtd/). Figure 2a shows the optical intensity distribution at the *x*–*y* plane in the middle of the 250-nm-thick silicon slab (*z* = 125 nm). The input light is centered at the wavelength of 1550 nm along the +*x* direction. The designed metalens is 11 µm wide in the *y* direction, with a focusing length of 25 µm and a spot size of 1.07 μm. The spot size is marked as the full width half maximum (FWHM) in the cross section of the mode profile (inset of Fig. 2a). Figure 2b shows the detailed in-plane electric field distribution of **E**_**y**_ across the metalens. With a gradient varying phase shift, the interference between the transmitted wave ‘bends’ the off-axis light toward the central axis. The focusing length of the metalens can be controlled by varying the gradience of the slot length. The simulated focal length is 6.7% shorter than the analytically design, due to the finite phase shift between adjacent slots. The focusing lengths can be adjusted from a few µm to sub-mm by controlling the step size of slot length in *y* direction. Figure 2c plots the focusing efficiency, transmission, and spot size versus focal lengths, indicating a trade-off between the focusing efficiency and spot size. At a fixed lattice constant, a short focusing length demands a larger gradient of the slot length, which increases the deviation of the transmitted wavefront compared with that of the ideal lens. As the focusing length reduces from 28 to 8 µm, the focusing efficiency reduces from 80% to 40%. The focusing efficiency is defined as the fraction of the incident light that passes through a rectangular aperture at the focal plane, with its width equals to three times the spot size and a height of 0.5 μm. For metalens with focusing lengths longer than 25 µm, a maximum focusing efficiency of 79% can be achieved (Fig. 2c). The spot size can be reduced to 0.4 µm at a shorter focusing length of 8 µm.

We also verify the broadband operation of the on-chip metalens. The focusing efficiency is above 74% and transmission is above 88% within a 200 nm bandwidth centered at 1550 nm (Fig. 2d). The focusing efficiency and transmission of the metalens varies less than 3% across the whole wavelength range. The spot size is about 1.08 µm in the wavelength range from 1550 to 1650 nm, and slightly increases (~0.05 µm) at shorter wavelengths (near 1450 nm). The strong light confinement in the slots minimizes the material dispersion in the HCTA (Fig. 1b, c), as silicon dioxide’s chromatic dispersion is an order of magnitude smaller than silicon. The broadband low dispersion (Supplementary Figs 2 and  3) is attributed to the geometric dispersion (with details discussed in Supplementary note 3). At oblique incidence, the metalens retains its transmission efficiency of 90% and focusing efficiency of 82% as the tilting angle is tuned from −5^o^ to 5^o^. The focusing efficiency then gradually decreases down to 65% at 20^o^ incidence angle, along with an increase in spot size (Fig. 2e).

**Fig. 3:**
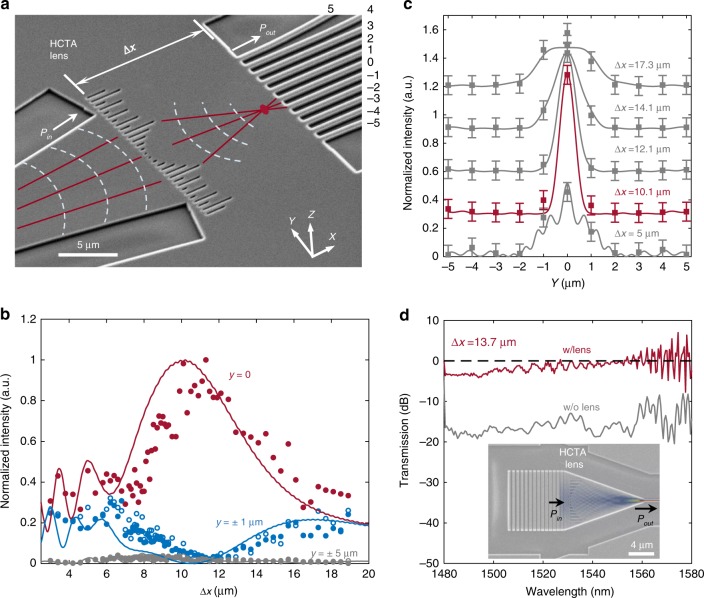
Demonstration of on-chip low loss metalens for ultracompact taper. **a** Scanning electron microscope (SEM) image of an on-chip metalens with a focusing length of 8 μm, object distance of 44 µm, and image distance of 10.1 μm. Eleven single mode waveguides are placed on the output plane for mapping the spatial distribution of light. **b** Comparison of the simulated (solid lines) and measured intensity profile (filled and empty circles stand for the positive and negative coordinates respectively) along the optical axis. **c** The mode profile evolvement with Δ*x*, and the black line is the profile at the focusing plane. The error bars represent the standard deviation (s.d.) for three measurements. **d** Transmission spectra of a 13.7-μm-long taper with and without metalens, after normalization to a grating coupler with a 250-μm long taper. Inset: SEM image of the ultracompact metalens taper connecting a grating coupler (width of 11 μm) and single mode waveguide (width of 0.5 μm) superimposed with the simulated intensity distribution

The numerically evaluated performance of the on-chip metalens is then experimentally verified. The on-chip mode conversion is mapped in both the *x* and *y* directions. In the *y* direction, 11 waveguides are parallelly placed on the output plane to obtain the optical intensity distribution. Their center-to-center distances are set at 1 µm to avoid interference. The optical field variation in the *x* direction is sampled by 67 devices with their metalens and output plane distance (*Δx*) arranged from 3 to 18.9 μm. Figure 3a shows a scanning electron microscope (SEM) image of a device. The focal length *f* of the metalens under test is designed to be 8 μm. A 44-µm-long exponential taper guides the input light from a single mode waveguide (0.5 µm width) to the metasurface with a diameter of 11 µm. The metalens converges the beam width down to a spot size of 0.75 μm (FWHM) at the image distance of $\Delta x = 10.1\;{\mathrm{\mu m}}$ (Fig. 3b–c). The simulated and measured optical intensity distribution along the *x*-axis at different *y* positions are compared in Fig. 3b. The measured cross-sectional light intensity profiles (dots in Fig. 3c) align well with the simulated profiles (curves in Fig. 3c). The beam FWHM decreases to its minimum at ${\mathrm{\Delta }}x = 10.1\;{\mathrm{\mu m}}$, where the light intensity increases an order of magnitude compared with the input wave.

With metalens assisted light focusing, a 13.7-μm-long linear taper can efficiently convert the mode from an 11-μm width down to a 0.5 µm width (Inset of Fig. 3d). Figure 3d compares the transmission spectra of the 13.7-μm-long taper with and without the metalens. The metalens is placed beside the grating coupler, as shown in the SEM image of the device (inset of Fig. 3d). The measured insertion loss of the metalens is less than 0.8 dB in the C band and increases to 2 dB in the S-band, which is comparable to the simulated results (transmission above 79% in the wavelength band from 1480 to 1580 nm). An extra 18 dB loss is observed for the same device design without a metalens (gray curve in Fig. 3d). This result experimentally demonstrates the broadband high transmission of the metalens, a critical feature required for multi-stage system integration.

### Mathematical operation with cascaded meta-system

**Fig. 4:**
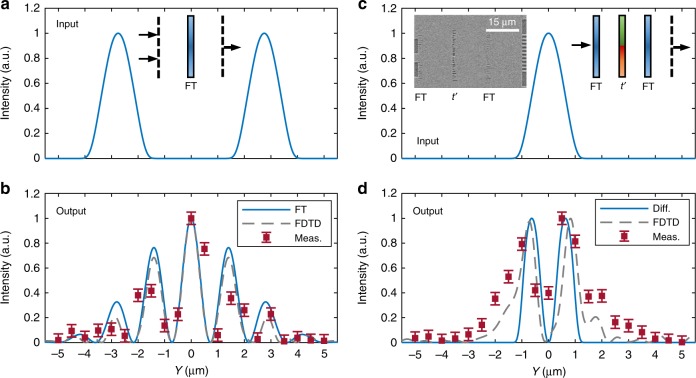
Mathematical operation with cascaded HCTA. **a** The input and **b** output of an on-chip Fourier Transform (FT) system. The inset of **a** shows the schematic design of the system (30-μm long). The error bars represent the s.d. for three measurements. **c** The input and **d** output of an on-chip differentiator (45-μm long). The insets of **c** show the schematic diagram and correspondent layout of the device. The measurement result (red squares in **b,** **d**) is compared with the FDTD simulated profile (gray dashed curve) and analytical results (blue solid curve). The error bars represent the s.d. for three measurements

Compared with conventional free-space optical information processing metasystems, the advanced lithography technique allows for easy alignment of the on-chip metasurface for a cascaded multilayer system with precise spacings. Here we use the designed metalens for implementing spatial Fourier transformations (FT). For a proof-of-concept demonstration, a device with 2 input ports and 11 output ports are fabricated on an SOI substrate (Supplementary Fig. 4a, b). The input and output planes are placed on the focal plane of the metalens (Inset of Fig. 4a). On the input port, light is coupled from single mode waveguides to the slab (profile is shown in Fig. 4a). On the output plane, the optical field distribution is sampled by densely spaced single mode waveguides with 1 μm center-to-center spacing. The transmitted power from those ports is measured and compared with the numerical and analytical results (Fig. 4b). The output intensity distribution can be directly converted to the FT of input intensity profile, by converting the *y* axis to spatial frequency $\left( {\xi = y/(\lambda f)} \right)$. Here, *λ* is the wavelength in the slab waveguide and *f* is the focal length. The design principle of the FT system is introduced in Supplementary Note 4.

Based on the FT element, the spatial differentiation system is then developed to perform the spatial differentiation on the input signal (Supplementary Note 4). The differentiation meta-system is composed of three layers (Insets of Fig. 4c). The first and third layers of the meta-system are metalens, and the second layer is an HCTA mask layer with a specific transmission coefficient $t\left( y \right) \propto \left[ {jy \cdot {\mathrm{exp}}\left( { - j\pi \frac{{y^2}}{{\lambda f}}} \right)} \right]$ (Supplementary Fig. 4c, d). The spatially varying transmission coefficient is programed onto the middle HCTA mask layer by varying the width and the length of the slot (Fig. 1d, e). As shown in the left inset of Fig. 4c, the three stage system only has a footprint of 15 µm by 45 µm. A 2.5 µm wide waveguide guides light to the input plane of the metasystem. On the output plane, 11 waveguides are parallelly connected with a 1-µm center-to-center distance (Fig. 4c). Combining results from two identical devices with output waveguides arrays shifted 0.5 µm long the *y* direction, a spatial resolution of 0.5 µm can be achieved (red squares in Fig. 4b, d). The measured spatial spectra align with the numerically simulated and analytical results (Fig. 4d). We should note that the output has reversed coordinates, as the FT is applied twice. More results of Fourier transform and spatial differentiation can be found in Supplementary Figs. 6–8. It is noted that the focusing efficiency and focal distance vary less than 9% at incident angle from −16^o^ to 16^o^ (Fig. 1e and Supplementary Fig. 9). In our meta-systems (Fig. 4), the incident angle is confined within ±16°, resulting in accurate FT (Supplementary Note 5 and Supplementary Fig. 10).

With lithography defined metasurface orientation and spacing, the on-chip meta-system possesses a small footprint and high stability. We also verify the scalability and Foundry compatibility of our metasystem. Supplementary Note 4 includes results of the AIM photonics manufactured metasystems, including FT and spatial differentiation functions (Supplementary Fig. 5).

## Discussion

Here we presented a foundry fabrication compatible, ultracompact metasurface designed to achieve on-chip wavefront transformation with low insertion loss and broadband operation. An HCTA based metalens is analytically designed, numerically verified and experimentally demonstrated. The 1D metalens has a numerical aperture up to 2.14, which can focus light to within 10 µm with less than 1 dB loss. It can significantly shrink the length of tapers for mode size conversion. The spatial FT of the input signal is achieved by placing the input and output on the focal plane of a metalens. More complicated computational tasks based on FT can be performed by cascading multiple layers of HCTA. As an example, we demonstrate a three-layer metasystem for performing spatial differentiation.

The experimental demonstration of the functional HCTAs opens new directions for on-chip diffractive optical systems, which is distinguished from conventional waveguide based integrated photonic devices. The on-chip metasurface can be integrated with multimode waveguides^38,39^, to perform mode transformation in mode division multiplexing systems. Also, the 1D metasurface design enables many novel on-chip systems with low power consumption and ultracompact dimension, including diffractive optical computational circuits, on-chip spectrometers and light detection and ranging devices.

## Methods

### Device fabrication

The on-chip metasurface is fabricated on an SOI (100) substrate from Soitec, with the 250 nm device layer on 3 μm silicon dioxide layer. The designed patterns (e.g., HCTA, waveguides, grating couplers) are defined in CSAR 6200.09 positive resist using a Vistec EBPG5200 electron beam lithography system with 100 kV acceleration voltage, followed by resist development and a single step dry etch procedures. A 300-nm-thick silicon dioxide protection layer is deposited on the device layer by plasma-enhanced chemical vapor deposition. Part of the metasystem structure is manufactured through a multi-project wafer run at The American Institute for Manufacturing Integrated Photonics (AIM Photonics).

### Optical measurements

Continuous-wave tunable semiconductor lasers (AQ4321D) with a polarization controller is used for launching light onto the chip. The fiber-grating coupler loss is optimized to be 5 dB per input/output facet. The propagation loss in the channel waveguides is less than 1 dB. The output is monitored by a Newport InGaAs photodiode (818-IG-L-FC/DB) and an optical power meter (1830-R-GPIB).

### Numerical simulations

A three-dimensional finite-difference-time-domain method (http://www.lumerical.com/tcad-products/fdtd/) is used to simulate the optical field distribution and transmission spectra of the periodic HCTA, metalens, and the meta-systems. The conformal mesh with spatial resolution less than 1/10 of the smallest feature size is applied. For calculating the transmission of HCTAs, a *y–z* plane monitor is placed at *Δx* = 3 μm to collect the transmitted power. *Δx* is the distance to the left aligned side of the HCTA.

## Supplementary information

Supplementary Information

## Data Availability

The data that support the findings of this study are available from the corresponding author upon reasonable request.
