# Supplementary material for: On-chip wavefront shaping with dielectric metasurface
Source: Nat Commun. 2019 Aug 7;10:3547. doi: 10.1038/s41467-019-11578-y (PMC6686019; doi:10.1038/s41467-019-11578-y)
Supplement: Supplementary file 1 — Supplementary Information [file 41467_2019_11578_MOESM1_ESM.pdf]

Supplementary Material for

On-chip wavefront shaping with dielectric metasurface

Zi et al.

Supplementary Material for

On-chip wavefront shaping with dielectric metasurface

Zi Wang, Tiantian Li, Anishkumar Soman, Dun Mao, Thomas Kananen and Tingyi Gu*
Department of Electrical and Computer Engineering, University of Delaware, Newark, DE 19711, USA
*Email: tingyigu@udel.edu

Supplementary note 1: Performance comparison of the metalens

Supplementary Table 1 compares the performance matrix of integrated metalens (processing optical signals in plane). The summary shows that our design has a smaller footprint, lower loss, simpler fabrication process, and larger critical dimension.

Supplementary Table 1 | Performance comparison of on-chip metalens

Lens type	Device length	Critical dimension	Insertion Loss (dB)	Bandwidth	System/Circuit demonstration
1D voids on SOI (This work)	2.4 µm	140 nm	<0.8	>200 nm	Y
2D Au array [S1]	4 µm	50 nm	2-4	100 nm	N
1D longitutional WG array on SOI [S2]	20 µm	100 nm	NA	NA	N

WG: Waveguide

Supplementary note 2: 1D HCTA design

In the design of the 1D HCTA, we focus on achieving two factors: reducing the wavefront distortion and maximizing the transmission. As a plane wave centered at 1550 nm passing through the interface from the slot to silicon slab, the phase shift along the direction of light propagation 'bends' on the interface (blue line in Supplementary Figure 1a). The electric field intensity is ploted as the red curve in Supplementary Figure 1a.

The effective refractive index of the periodic slot array can be approximated by the effective-medium theory (EMT) [S3-S4]. EMT defines the subwavelength periodic grating structure as a uniaxial crystal [S4], with effective permittivity given as:

$$\varepsilon_{\perp}^{(2)} = \varepsilon_{\perp}^{(0)}\left[1+\frac{\pi^2}{3}\left(\frac{\Lambda}{\lambda}\right)^2 f^2(1-f)^2\frac{\left(\varepsilon_{Si\perp}-\varepsilon_{SiO_2}\right)^2}{\varepsilon_0\varepsilon_{\perp}^{(0)}}\right], \qquad (S-1)$$

when the electrical field E is perpendicular to the grating vector K, and by

$$\varepsilon_{\parallel}^{(2)} = \varepsilon_{\parallel}^{(0)}\left[1+\frac{\pi^2}{3}\left(\frac{\Lambda}{\lambda}\right)^2 f^2(1-f)^2\left(\varepsilon_{Si\parallel}-\varepsilon_{SiO_2}\right)^2\frac{\varepsilon_{\perp}^{(0)}}{\varepsilon_0}\left(\frac{\varepsilon_{\parallel}^{(0)}}{\varepsilon_{Si\parallel}\varepsilon_{SiO_2}}\right)^2\right], \qquad (S-2)$$

when E is parallel to K, where Λ is the period of the grating, λ is the wavelength in free space, $\varepsilon_{Si\perp}$ and $\varepsilon_{Si\parallel}$ are the effective permittivity of the TM wave and TE wave in silicon slab. ε_{SiO_2} is the relative permittivity of the silicon dioxide within the slot. f is the filling factor of the slots given by $f = w_{slot}/a$, where w_{slot} is the slot width and a is the lattice constant of the periodic slots. In Eqs. (S-1) and (S-2), $\varepsilon_{\perp}^{(0)}$ and $\varepsilon_{\parallel}^{(0)}$ stand for the zeroth-order approximations of the effective permittivity which are given by:

$$\varepsilon_{\perp}^{(0)} = f\varepsilon_{SiO2} + (1-f)\varepsilon_{Si\perp}, \qquad (S-3)$$

$$\left(\varepsilon_{\parallel}^{(0)}\right)^{-1} = f\left(\varepsilon_{SiO_2}\right)^{-1} + (1-f)(\varepsilon_{Si\parallel})^{-1}. \qquad (S-4)$$

We compare the EMT (square root of the second order approximation of the effective permittivity) derived effective index (n) of the HCTA to the FDTD simulation results (Fig. S1b). The alignment between the estimated (EMT) and numerically calculated result (FDTD) validate design principles. Alignment between the two results confirms the accuracy of effective-medium theory (especially as slot width smaller than 0.2μm). The effective media theory based effective index estimation is much faster and easier than FDTD, and thus can be used for the design of more complicated cascaded HCTA systems.

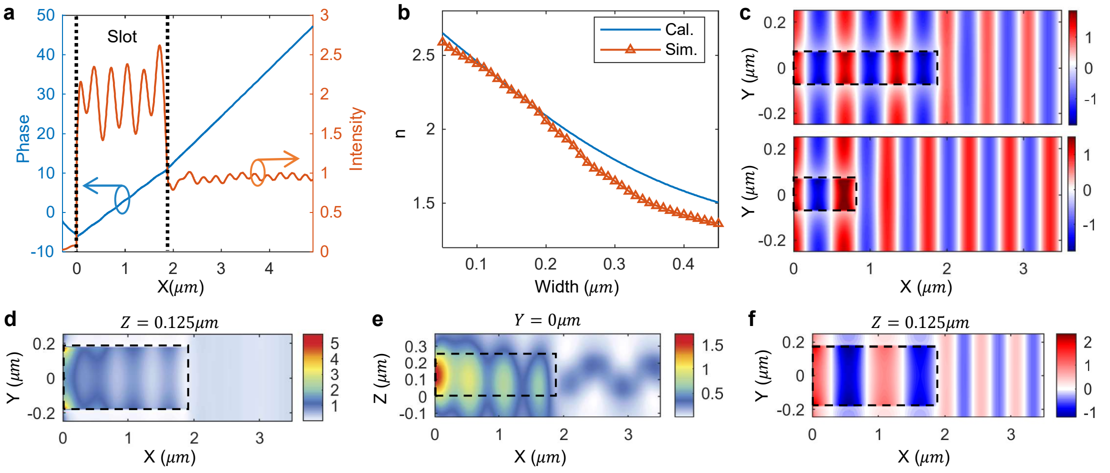

Supplementary Figure 1. Design principle of the lossless HCTA. (a) The simulated phase and intensity distribution along the x-axis at the center line of the slot ($y = 0,\ z = 0.125\ \mu m$).

(b) The effective refractive index of the 1D HCTA verus slot width. Blue curve: effective medium theory calculation; Orange curve: numerical simulation. **(c)** The simulated TE field distribution of the x-y plane at $z = 0.125$ μm with the different lengths of the slot. **(d) and (e)** The simulated TE intensity distribution of the x-y plane and x-z plane with the slot's width of 360 nm. **(f)** The simulated TE field distribution of the x-y plane at $z = 0.125$ μm with the slot's width of 360 nm.

Fig. S1c shows the example of electric field distribution within 140 nm wide slots. As a plane wave passing through the 1D HCTA, the phase shift at X = l_{max} is defined as (assuming the left edges of the slots are aligned at X = 0):

$$\Delta\phi = k_{slot} l + k_{slab}(l_{max} - l), \qquad (S-5)$$

where $k_{slot/slab} = n_{slot/slab} * k_0$ is the effective wave number of the slot region/silicon slab, l is the length of the slot, and l_{max} is the length of the HCTA along x direction. k_0 is the wavenumber in free space. n_{slot} can be estimated from Fig. S1b, given the slot width. n_{slab} is 2.9 for the 250 nm thick silicon slab embedded in the silicon oxide claddings. π phase shift can be achieved with slot length difference of 1.2 μm.

The transmission of HCTA reduce dramatically as the slot width wider than 200 nm. Fig. S1d-f show the electric field intensity in XY and YZ planes and amplitude in XY plane at the slot width of 360 nm. Strong reflection/scattering leads to reduction of the transmitted light.

Supplementary note 3: Dispersion of the on-chip metalens

Here we firstly analytically discuss the origin of low dispersion in the 1D metalens.

In a metlens, the *y* axis dependent phase shift of transmitted wave is [S5]:

$$\phi(y) = \omega n_{slab} * \left(\frac{f - \sqrt{f^2 + y^2}}{c}\right) = \omega n_{slab} * T(y), \qquad (S-6)$$

where ω is the angular frequency of the incident wave; c is the speed of light; $T(y)$ is a frequency independent factor. $T(y)$ controls the dispersion of the metalens[S5].

Eqs. S-5 can be wrote as:

$$\Delta\phi = \omega n_{slab} * \left(\frac{n_{slot}}{n_{slab}} * \frac{l}{c} + \frac{l_{max} - l}{c}\right) = \omega n_{slab} * T'(\omega, y), \qquad (S-7)$$

where ω is the angular frequency of the incident wave, c is the speed of light in free space,

$T^{'}(\omega, y) = \left(\frac{n_{slot}}{n_{slab}} * \frac{l}{c} + \frac{l_{max} - l}{c}\right)$ varies less than 2% from 1450 nm to 1650 nm, indicating low dispersion of the designed metalens.

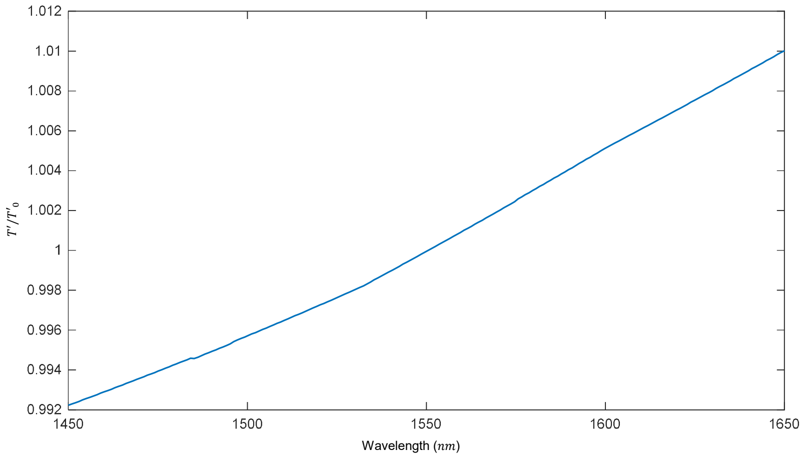

Supplementary Figure 2. Normalized disperison coefficient of the on-chip metalens from 1450 nm to 1650 nm. T_0*'* is the dispersion coefficient at 1550 nm.

The analytical discussion is then verified through numerical simulation. S3a shows the simulated intensity distribution of the on-chip lens with paralell incident light along *x* axis. The simulation shows that for a lens optimized for wavelength $\lambda = 1550$ nm, the focal length of 25 μm increases to 27.9 μm at 1450 nm, and decreases to 22.9 μm at 1650 nm. The optical field distribution in yz plane at $x = 25$ μm vary little for wavelength range across S, C and L bands (Fig. S3b).

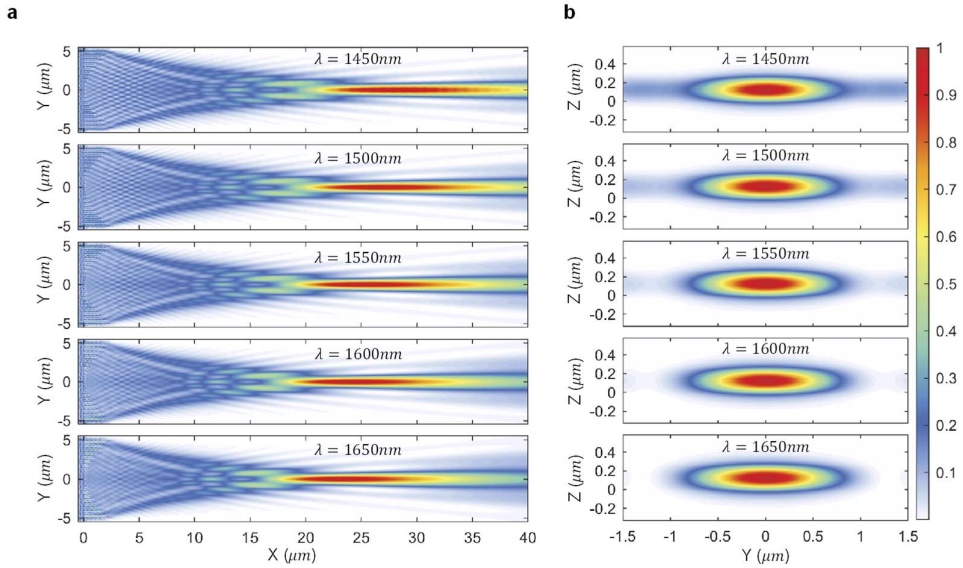

Supplementary Figure 3. Dispersion property of the on-chip metalens. The figure shows

the simulated focusing effect with 5 different wavelengths in **(a)** x-y plane and **(b)** y-z plane at $x = 25$ μm.

Supplementary note 4: On-chip meta-system: design, fabrication and measurement

Supplementary note 4.1: Design principle

With an input signal of *f(x,y)*, the output function of a two dimensional metalens at its focal plane is given by [S6]:

$$g(x,y) = c' exp\left[j\pi \frac{(x^2+y^2)(f-d)}{\lambda f^2}\right] \mathcal{F}\left(\frac{x}{\lambda f}, \frac{y}{\lambda f}\right), \quad (S-8)$$

Where j is the imaginary unit; (x, y) are the coordinates on the focal plane; c' is a constant ; f is the focal length of the lens; d is the object distance, or the distance between the input plane and the lens; λ is the wavelength; $\mathcal{F}$ stands for Fourier transform of the input signal. For one dimensional lens, Eqs. (S-8) can be simplified as:

$$g(y) = c' exp\left[j\pi \frac{y^2(f-d)}{\lambda f^2}\right] \mathcal{F}\left(\frac{y}{\lambda f}\right). \quad (S-9)$$

At object distance *d* = 0, Eqs. S-9 becomes:

$$g(y) = c' exp\left(j\pi \frac{y^2}{\lambda f}\right) \mathcal{F}\left(\frac{y}{\lambda f}\right). \quad (S-10)$$

Based on the Fourier transform property of the metalens, we construct a three-layers meta-system to perform a spatial differentiation (Fig. 4a). The input signal *f*(*y*) can be expressed as the inverse Fourier transform form [S7]:

$$f(y) = \int \mathcal{F}(k_y) e^{jk_y y} dk_y. \quad (S-11)$$

Differentiation of the *f(y)* can be expressed as:

$$\frac{df(y)}{dy} = jk_y \int \mathcal{F}(k_y) e^{jk_y y} dk_y. \quad (S-12)$$

Comparing Eqs. S-12 and metalens output (Eqs. S-10), we conclude that at the focal plane of the metalens, an additional metasurface with a transmission coefficient $\propto (jk_y)$ and phase shift of $exp\left(-j\pi \frac{y^2}{\lambda f}\right)$ need to be introduced in Eqs. (S-10). After adding another layer of metalens, the output function $\propto \frac{df(y)}{dy}$.

Supplementary note 4.2: Device fabrication

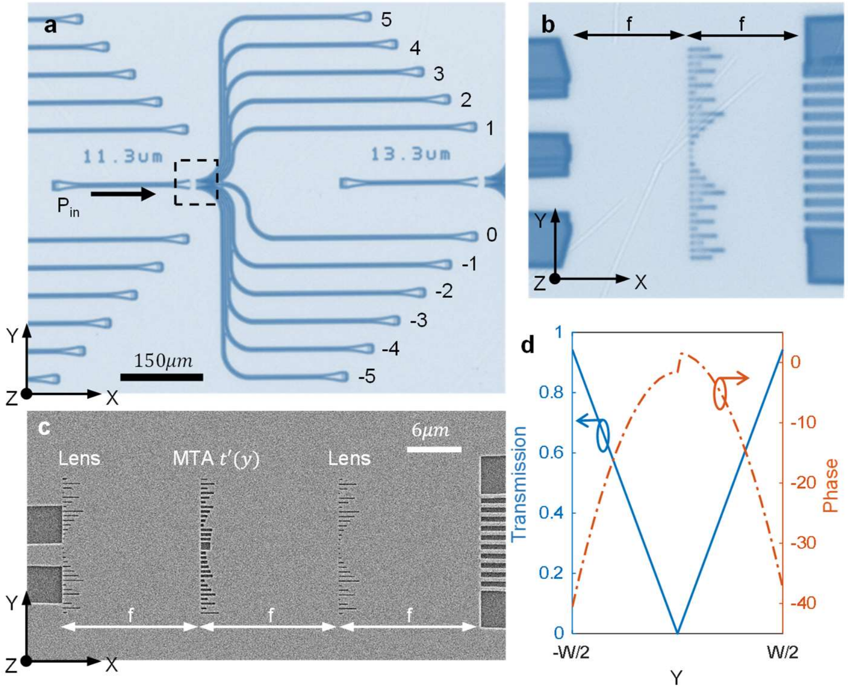

Supplementary Figure 4. Device fabricated at University cleanroom. (a) Top view of an on-chip metalens structure. The input light is fed through the waveguide on the left. As the light passed through the microlens, the output is collected through single-mode waveguides at the different spatial location. **(b)** The optical microscope image of the Fourier transform structure based on the metalens. **(c)** The SEM image of the three-layer meta-system that can do the spatial differentiation of the input signal. **(d)** The required transmission and phase change of the second layer HCTA in the spatial differentiation meta-system.

To experimentally varify the evolvement of in-plane beam profiel, we designed an "octopus-shaped" structure to map the optical field distribution in *x*-*y* plane as shown in Fig. S4a. The input/output light is coupled in/ out of the diffractive components by grating couplers, and the optical path way from the output plane to grating couplers are identical to minimize any extra loss variation. Fig. S4b shows the structure to verify the Fourier transform property of the on-chip metalens. The distance between each plane (input plane, metalens plane, and output plane) equals the focal length of the metalens f. When the distance d in Eqs. S-9 equals to f, we can get Fourier transform of the input at the back focal plane.

Fig S4c shows the SEM image a three-layer HCTA performing the spatial differentiation.

The first and the third layers are metalens, and second and the output waveguides are placed on their focal plane . The coded transmission coefficient of the second layer is shown in Fig. S4d. We should note that since we can't reach 0 transmission with the critical dimension of $140\mu m$, we just combined the two slots in the center to a bigger slot to reach zero transmission.

Larger scale devices are fabricated at The American Institute for Manufacturing Integrated Photonics (AIM Photonics). Fig. S5 shows a three-layer meta-system fabricated by AIM Photonics. The inputs were generated by three cascaded 2×2 Multi-mode interferometers (MMI) to have a phase difference of π between two input ports.

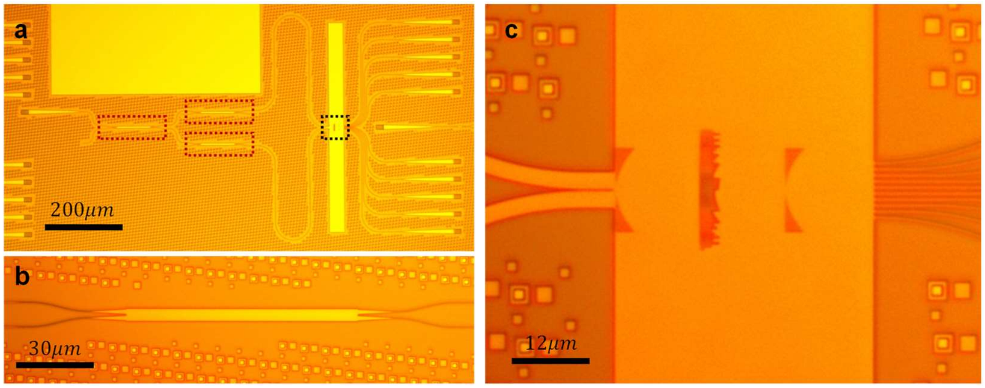

Supplementary Figure 5. Optical microscope image of the on-chip three-layer meta-system fabricated at a CMOS foundry (AIM Photonics [S8]). (a) The layout of the meta-system which can perform the spatial differentiation of the input signal. The zoomed in images of the red dotted box and black dotted box are shown in **(b)** and **(c)**. **(b)** The optical microscope image of a 2×2 Multi-mode interferometer (MMI) which can provide a 0.5π phase difference between the two output ports. **(c)** The optical microscope image of the three-layer meta-system.

Supplementary note 4.3: Experimental demonstration

Here we measure the fabricated devices for fourier transform and differention, and compared with analytical calculation and numerical analysis (Fig. S5-S8).

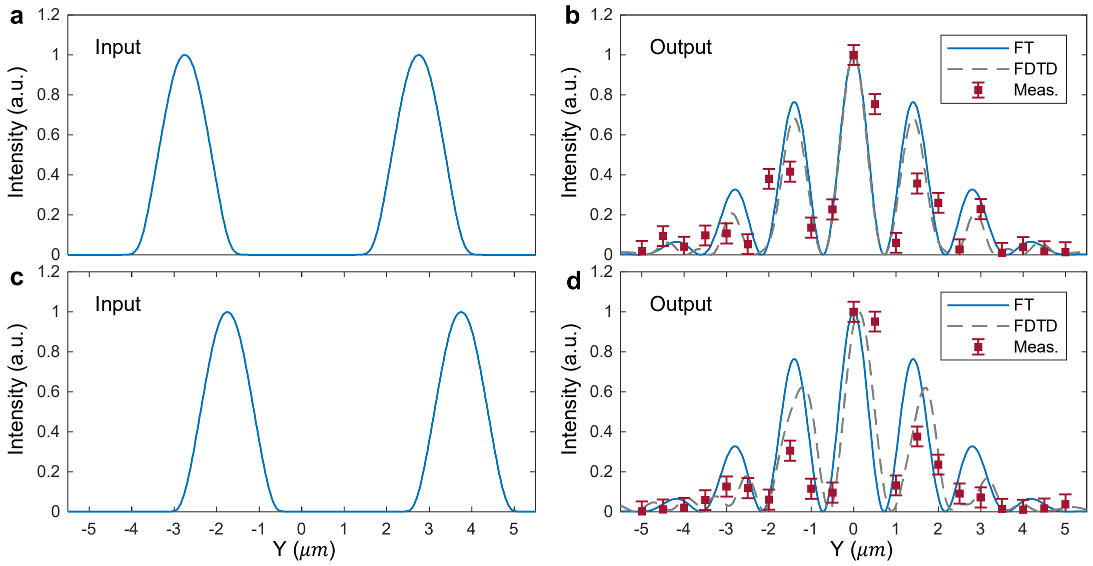

Supplementary Figure 6. Fourier transform with the metalens. (a) The input and **(b)** output of the on-chip Fourier Transform (FT) system (same as Fig. 4a and Fig. 4b). The error bars represent the standard deviation (s.d.) for three measurements. **(c)** The input and **(d)** output of the same FT system with a 1-μm offset along the y-axis of the input. The measurement result (red squares in b and d) is compared with the FDTD simulated profile (grey dashed curve) and analytical results (blue solid curve). The error bars represent the s.d. for three measurements.

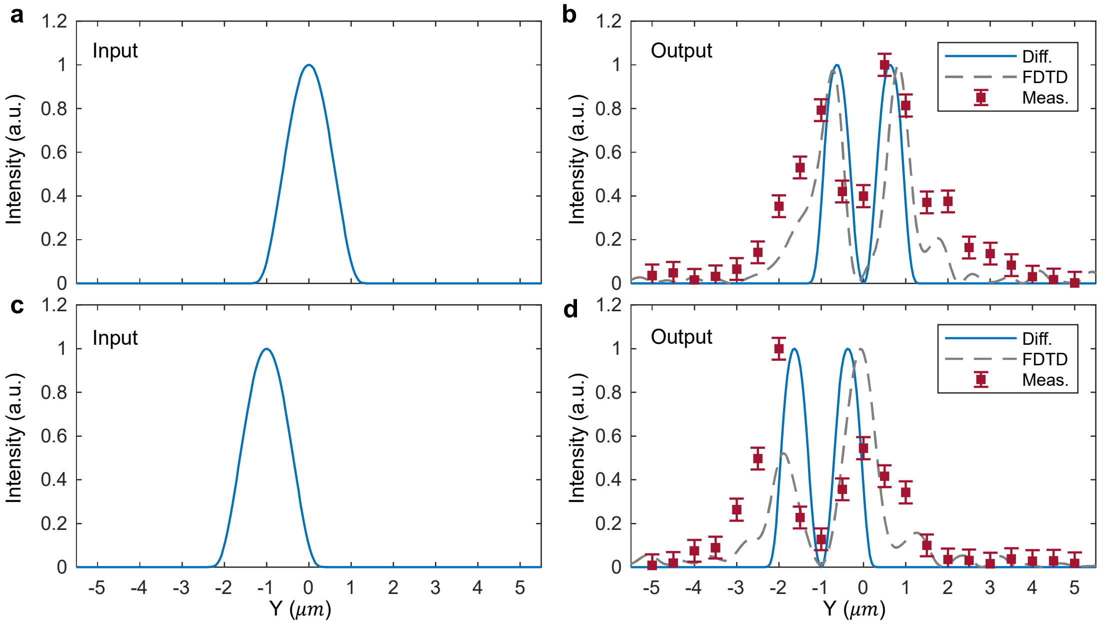

Supplementary Figure 7. The differentiation operation of one input. (a) The input and **(b)** output of the on-chip spatial differentiation system (same as Fig. 4c and Fig. 4d). The error

bars represent the s.d. for three measurements. **(c)** The input and **(d)** output of the same differentiation system with a 1-μm offset along the y-axis of the input. The measurement result (red squares in b and d) is compared with the FDTD simulated profile (grey dashed curve) and analytical results (blue solid curve). The error bars represent the s.d. for three measurements.

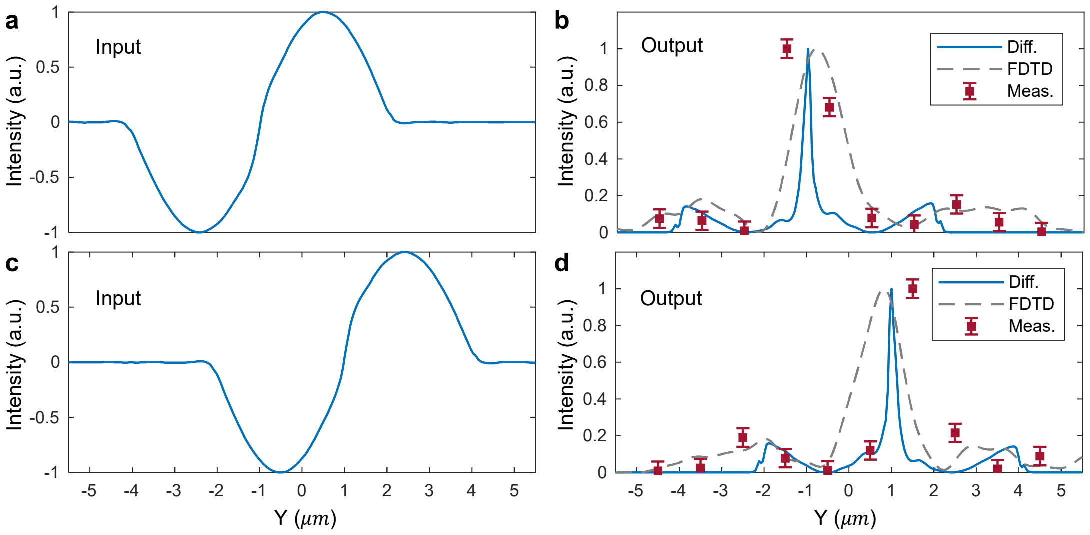

Supplementary Figure 8. The differentiation operation of two inputs with π phase difference. (a) The input and **(b)** output of the on-chip spatial differentiation system. The error bars represent the s.d. for three measurements. **(c)** The input and **(d)** output of the same differentiation system with an 2-μm offset along the y axis of the input. The inputs were generated by three 2×2 MMI which provide two input ports that have a π phase difference. The measurement result (red squares in b and d) is compared with the FDTD simulated profile (grey dashed curve) and analytical results (blue solid curve). The error bars represent the s.d. for three measurements.

Supplementary note 5: Angle dependent distortion on Fourier transform

The metalens is capable of focusing light at both normal and oblique incidence, however, the focusing behavior varies with incident angle. At oblique incidence, the focusing efficiency reduces and the focal length is shortened (Fig. S9). The angle dependence of the focal length and focusing efficiency is little at small incident angles (0~10°), and slightly increases at large incident angle (10~20°). In Fig. S9, we highlighted the acceptable distance for light focusing/FT with little distortions.

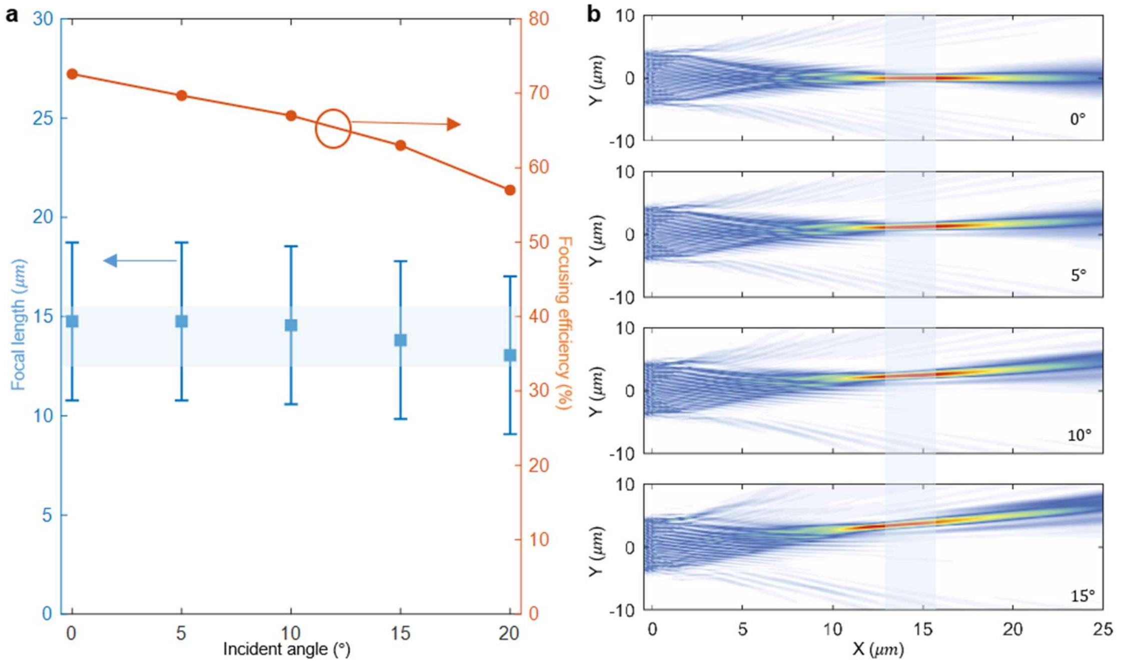

Supplementary Figure 9. The dependence of focusing behavior on incident angle for a metalens with 14.8 μm focal length. (a) Focal length and focusing efficiency versus incident angle. The error bars mark the upper and lower bound of focusing depth (3.98μm). **(b)** The simulated intensity profile with different incident angle. The shaded blue areas in (a) and (b) highlight the acceptable distance of focusing.

In FT, the angle of the wave vector related to the spatial frequencies as $\theta_y = sin^{-1}(\lambda_0 v_y / n_{slab})$ [S6], where θ_y is the incident angle, λ_0 is the wavelength in free space, n_{slab} is the effective index of silicon slab and v_y is the spatial frequency (Fig. S10a). In Fig. 4, the spatial frequencies of three peaks are $0.176\mu m^{-1}$, $0.352\mu m^{-1}$, $0.528\mu m^{-1}$ respectively, corresponding to the incident angles of $5.35°$, $10.76°$ and $16.26°$ (marked as red triangles in Fig. S10c). For a metalens with 14.8 μm focal length, its focusing efficiency reduces 9% and the focal length shifts near 0.9 μm as the incident angle is set at 16° (Fig. S9). The distortion caused by the metalens has limited influence on the Fourier transform pattern.

Also, we want to emphasize that our metalens has similar capabilities for FT compared to metalens for free space optics. The implementation in silicon photonic platform has benefits that the linear relation range between spatial frequency (v_y) and angle is extended by 2.92 (n_{slab}) times. A 1D metalens in free space is demonstrated to operate with incidence angle of $0 - 60°$ for distortionless FT [S9]. The correspondent spatial frequency for $60°$ is $0.56\mu m^{-1}$ at

1550nm, which can be achieved in silicon photonic platform with 17° incidence angle as shown in Fig. S10a.

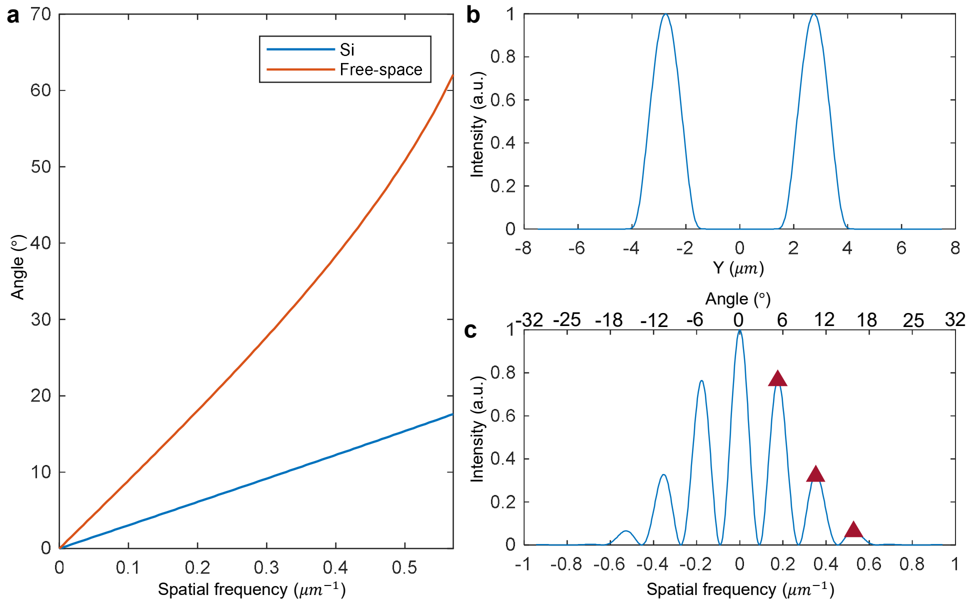

Supplementary Figure 10. Incident angle and Fourier transform pattern versus the spatial frequency. (a) The incident angle versus spatial frequency in free space and silicon slab waveguide. **(b)** The input and **(c)** Fourier transform of the input signal. The spatial frequencies of three peaks (marked as red triangles) are $0.176\mu m^{-1}$, $0.352\mu m^{-1}$, $0.528\mu m^{-1}$ respectively.

Supplementary References

[1] Fan, Y., Le Roux, X., Korovin, A., Lupu, A., & de Lustrac, A. Integrated 2D-graded index plasmonic lens on a silicon waveguide for operation in the near infrared domain. *ACS nano* **11,** 4599-4605 (2017).

[2] Levy, U., Abashin, M., Ikeda, K., Krishnamoorthy, A., Cunningham, J. & Fainman, Y. Inhomogenous dielectric metamaterials with space-variant polarizability. *Phys. Rev. Lett.* **98,** 243901 (2007).

[3] Rytov, S. Electromagnetic properties of a finely stratified medium. *Soviet Physics JEPT* **2,** 466-475 (1956).

[4] Raguin, D. H., & Morris, G. M. Antireflection structured surfaces for the infrared spectral region. *Appl. Opt.* **32,** 1154-1167 (1993).

[5] Arbabi, E., Arbabi, A., Kamali, S. M., Horie, Y., & Faraon, A. Controlling the sign of

chromatic dispersion in diffractive optics with dielectric metasurfaces. *Optica* **4,** 625-632 (2017).

[6] Saleh, B. E., & Teich, M. C. Fundamentals of photonics. (2007).

[7] Silva, A., Monticone, F., Castaldi, G., Galdi, V., Alù, A., & Engheta, N. Performing mathematical operations with metamaterials. *Science* **343,** 160-163 (2014).

[8] http://www.aimphotonics.com/

[9] Liu, W., Li, Z., Cheng, H., Tang, C., Li, J., Zhang, S., ... & Tian, J. Metasurface Enabled Wide-Angle Fourier Lens. *Adv. Mater.* **30,** 1706368 (2018)..
